# An overview of log likelihood ratio cost in forensic science – Where is it used and what values can we expect?

**DOI:** 10.1016/j.fsisyn.2024.100466

**Published:** 2024-04-17

**Authors:** Stijn van Lierop, Daniel Ramos, Marjan Sjerps, Rolf Ypma

**Affiliations:** a Netherlands Forensic Institute, Laan van Ypenburg 6, The Hague, 2497GB, the Netherlands; b AUDIAS Lab, Universidad Autonoma de Madrid, Escuela Politécnica Superior, Calle Francisco Tomàs y Valiente 11, 28049, Madrid, Spain; c University of Amsterdam, KdVI, PO Box 94248, Amsterdam, 1090 GE, the Netherlands

**Keywords:** *C*_*llr*_, Log-likelihood ratio cost, Automated likelihood ratio systems, Benchmarking review, Forensic evaluation, Forensic datasets, Performance of LR system

## Abstract

There is increasing support for reporting evidential strength as a likelihood ratio (LR) and increasing interest in (semi-)automated LR systems. The log-likelihood ratio cost (*C*_*llr*_) is a popular metric for such systems, penalizing misleading LRs further from 1 more. *C*_*llr*_ = 0 indicates perfection while *C*_*llr*_ = 1 indicates an uninformative system. However, beyond this, what constitutes a “good” *C*_*llr*_ is unclear.

Aiming to provide handles on when a *C*_*llr*_ is “good”, we studied 136 publications on (semi-)automated LR systems. Results show *C*_*llr*_ use heavily depends on the field, e.g., being absent in DNA analysis. Despite more publications on automated LR systems over time, the proportion reporting *C*_*llr*_ remains stable. Noticeably, *C*_*llr*_ values lack clear patterns and depend on the area, analysis and dataset.

As LR systems become more prevalent, comparing them becomes crucial. This is hampered by different studies using different datasets. We advocate using public benchmark datasets to advance the field.

## Highlights

• Publications describing (semi-)automated LR systems were studied.
• The number of publications on forensic automated likelihood ratio systems has been increasing since 2006.
• The proportion of publications reporting performance using the log likelihood ratio cost has remained relatively constant.
• No clear patterns were observed in *C*_*llr*_ values: these vary substantially between forensic analyses and datasets.

## 1 Introduction

In forensic science, support for using likelihood ratios (LRs) to assess the strength of forensic evidence has been growing [[1], [2], [3], [4], [5], [6], [7]]. Consequently, the issue of evaluating the performance of LR systems has been addressed in the recent past, focusing on their validation for use in casework [8]. In particular, several performance metrics have been proposed. The scientific community and forensic practitioners are notably utilizing the *Log Likelihood Ratio Cost*, also known as *C*_*llr*_, which was initially introduced in Ref. [9] within the context of likelihood-ratio-based speaker verifiers and subsequently adapted for forensic speaker recognition [10]. However, it is important to note that the use of the *C*_*llr*_ extends beyond speech-based systems and can be applied to any method that produces LRs.

The *C*_*llr*_ is defined as:

(1) $$C_{\mathrm{llr}}=\frac{1}{2}\cdot \left(\left[\frac{1}{N_{H_{1}}}\overset{N_{H_{1}}}{\underset{i}{\sum }}log_{2}\left(1+\frac{1}{LR_{H_{1_{i}}}}\right)\right]+\left[\frac{1}{N_{H_{2}}}\overset{N_{H_{2}}}{\underset{j}{\sum }}log_{2}\left(1+LR_{H_{2_{j}}}\right)\right]\right)$$

Here, $N_{H_{1}}$ is the number of samples for which *H*_1_ is true, $N_{H_{2}}$ is the number of samples for which *H*_2_ is true, *LR*_*H*__1_ are the LR values predicted by the system for samples where *H*_1_ is true, and *LR*_*H*__2_ are the LR values predicted by the system for samples where *H*_2_ is true. Hence, when we have a set of empirical LR values predicted by a certain LR system and the corresponding ground truth labels are available, we can calculate a *C*_*llr*_. Furthermore, the metric can be split into two parts, giving an indication of the error due to imperfect discrimination (‘do *H*_1_-true samples get a higher LR than *H*_2_-true samples?’) and imperfect calibration (‘is the value of the assigned LR correct, not under- or overstating the evidence?’). These two metrics can be calculated by applying the Pool Adjacent Violators (PAV) algorithm [11,12] on the evaluation set, mimicking ‘perfect’ calibration [9], and re-calculating *C*_*llr*_. The resulting value is taken as an assessment of discrimination (called *C*_*llr−min*_), the difference (*C*_*llr−cal*_ = *C*_*llr*_ − *C*_*llr−min*_) as an assessment of calibration [13,14].

Importantly, it is worth highlighting some advantages and drawbacks of the *C*_*llr*_ as a performance metric for LR systems. Among its advantages, the *C*_*llr*_ is a so-called ‘strictly proper scoring rule’, possessing favorable mathematical properties such as probabilistic and information-theoretical interpretation. As already mentioned, the metric provides an indication of both calibration and discriminating power of a method, allowing separate estimation of these two aspects of performance [15]. It thus considers not just whether an evaluation was misleading (i.e. the 10.13039/501100009319LR supports the wrong hypothesis), but also its associated evidential strength (e.g. misleading LR = 100 is worse than misleading LR = 2). It furthermore fosters incentives for forensic practitioners to offer accurate and truthful LRs, a critical aspect in a field where inaccurate or biased LRs can have significant implications for the criminal justice system, and imposes strong penalties for highly misleading LRs. In addition, the *C*_*llr*_ is a scalar that can be easily thresholded for validation, ensuring comparability between different systems, methods, and setups.

However, the *C*_*llr*_ also exhibits limitations. Primarily, as any empirical performance measure, it necessitates an empirical set of LRs, introducing challenges in database selection to generate these LRs. Ideally, these databases should resemble actual casework conditions, but such data is often limited, sometimes requiring a two-stage validation procedure using laboratory-collected data. Additionally, the *C*_*llr*_ is affected by small sample size effects, i.e., scarcity in empirically generated LRs, potentially leading to unreliable performance measurements. Data handling is a crucial concern in any validation process [8], as emphasized in many publications in the field [16]. Although certainly impacting *C*_*llr*_, these problems are common to all empirical evaluation metrics.

Another significant limitation of the *C*_*llr*_ is that, as a scalar, it provides a highly condensed statistic of model performance. In certain situations, further analysis may be beneficial to detect and correct model issues, leading to the proposal of alternative or specialized measures, as described below. The *C*_*llr*_ value can be split as a sum of *C*_*llr−cal*_ and *C*_*llr−min*_, describing the calibration error and discrimination error separately. While a large *C*_*llr−cal*_ value indicates an LR system overstates and/or understates the evidential strength, it does not tell us by how much and for which evidence class this tends to happen [17]. In addition, the *C*_*llr*_ value weighs the two different types of misleading evidence (misleadingly supporting *H*_1_ or *H*_2_) symmetrically, using a logarithmic scoring rule. One could debate whether or not this is appropriate in a forensic setting. Finally, the interpretation of the actual numerical value of the *C*_*llr*_ is not intuitive. Although lower is better and the value should be below 1, many researchers struggle with whether a value of say 0.3 can be considered ‘good’.

Various alternative performance metrics and representations have been proposed in the literature. Firstly, a more comprehensive picture can be obtained by looking at the full distribution of LRs under *H*_1_ and *H*_2_, e.g. using Tippett plots [8]. Similarly, the Empirical Cross-Entropy plot (ECE-plot) is often inspected as it generalizes the *C*_*llr*_ to unequal prior odds. Furthermore, many metrics exist that focus solely on either discriminating power or calibration. In the former category fall commonly used scalars such as accuracy or false positive rate, and representations as the Receiver Operating Characteristic curve (ROC) or its normalized version, the Detection Error Tradeoff (DET). ROC curves enable the computation of the Area Under the Curve (AUC) value, summarizing the discriminating power of a given method [15]. More recently, tools for assessing the calibration of LR systems have been proposed in the form of fiducial calibration discrepancies and corresponding plots [17], and the metric devPAV [18].

This paper focuses on the practical use of the *C*_*llr*_. We note an increasing number of scientific studies proposing LRs across various disciplines. Therefore, one may expect a corresponding increase in the use of the *C*_*llr*_. We conduct a systematic review of these studies, aiming to provide the forensic community with some intuition for what numerical values of the metric to expect or aim for. This may provide some guidance to the question as to how good a particular LR system performs beyond the anchors of *C*_*llr*_ = 0, indicating a perfect system, and *C*_*llr*_ = 1, indicating an uninformative system equivalent to a system that always returns LR = 1. Additionally, we give an overview of usage of this metric over time and across different forensic disciplines.

## 2 Materials and methods

We performed a literature search on October 31st, 2022 using a keyword search on the University of Amsterdam library and Scopus databases (Table 1). We took April 2006 as the start of the search range, since one of the first papers proposing the use of the *C*_*llr*_ metric to measure LR system performance was published in this month [9]. Hence, only English publications published between April 2006 and October 30th, 2022 were included in this review. In addition, we checked the references and citations of every selected paper to find additional relevant literature. We did this manually, but also performed an automated network search via the online tools Inciteful.xyz and connectedpapers.com. All articles were screened against several inclusion and exclusion criteria (Table 2).

**Table 1:** Keywords used for systematic literature search. All combinations of keyword 1, keyword 2 and optionally keyword 3 was used to query the University of Amsterdam and Scopus databases.

Keyword 1	Keyword 2	Keyword 3 (optional)
automated likelihood ratio system	forensic drug	investigation
cllr	forensic biometrics	analysis
cost-log likelihood ratio	blood spatter	evaluation
empirical cross entropy	forensic physics	identification
likelihood ratio	forensic chemistry	comparison
likelihood ratio model	forensic biology	validation
log likelihood ratio cost	digital forensics	individualisation
expert proficiency test	explosives	
automated system	microtraces	
score-based	forensic glass	
feature-based	forensic document	
	forensic text	
	gunshot residue	
	forensic handwriting	
	forensic image	
	forensic fiber	
	forensic hair	
	toolmark	
	footmark	
	forensic odontology	
	forensic firerarm	
	forensic fire	
	taphonomy	
	forensic toxicology	
	forensic soil	
	forensic paint	
	forensic plant	
	wildlife forensics	
	forensic anthropology	
	forensics	
	forensic dna	
	forensic speaker	
	forensic ballistics	
	forensic fingermark	
	forensic fingerprint	
	forensic audio	
	forensic	
	food forensics

**Table 2:** Inclusion and exclusion criteria used for systematic literature search.

Inclusion Criteria	Exclusion Criteria
Studies on forensic (semi-)automated LR systems.	Studies on human forensic experts that report LRs.
Studies published between April 2006 and October 30th, 2022.	Studies published before April 2006 or after October 30th, 2022.

If articles reported multiple *C*_*llr*_ values, the most forensically relevant *C*_*llr*_ was chosen, meaning the *C*_*llr*_ value that was the result of evaluation on data and conditions mostly resembling casework in the view of the authors. In case multiple models were evaluated on the same forensically relevant dataset, the *C*_*llr*_ of the best-performing model was selected. In cases where *C*_*llr*_ values were reported for multiple, all forensically relevant datasets, these *C*_*llr*_ values were included separately as they could be viewed as independent evaluations.

All data processing and visualization was done using Microsoft Excel 2022 [19] and the Pandas [20], NumPy [21], Matplotlib [22], Seaborn [23], SciPy [24] and NFI Likelihood Ratio [25] libraries in Python 3.10 [26]. All code for plotting and analysis as well as the dataset itself is available in the supplementary information.

## 3 Results

### 3.1 Proportion of publications reporting *C*_*llr*_

**Fig. 1:**
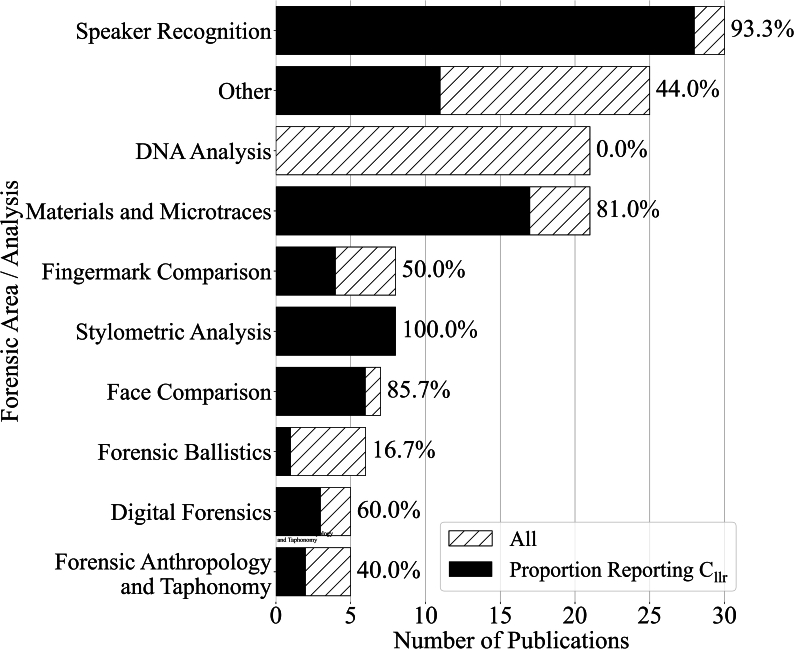
The number of publications on (semi-)automated likelihood ratio (LR) systems per forensic expertise area. The total number of publications per area is indicated by a dashed bar and the number of publications reporting one or more *C*_*llr*_ values per area is indicated by a solid bar. The bars are overlapped, not stacked. The areas are ordered based on absolute number of publications on (semi-)automated LR systems. The percentage on top of each bar indicates the proportion of publications reporting a *C*_*llr*_ value in that area. All publications from areas with less than 5 publications were pooled together in a single “Other” category.

In total, we found 136 publications on (semi-)automated LR systems [9,10,13,14,[27], [28], [29], [30], [31], [32], [33], [34], [35], [36], [37], [38], [39], [40], [41], [42], [43], [44], [45], [46], [47], [48], [49], [50], [51], [52], [53], [54], [55], [56], [57], [58], [59], [60], [61], [62], [63], [64], [65], [66], [67], [68], [69], [70], [71], [72], [73], [74], [75], [76], [77], [78], [79], [80], [81], [82], [83], [84], [85], [86], [87], [88], [89], [90], [91], [92], [93], [94], [95], [96], [97], [98], [99], [100], [101], [102], [103], [104], [105], [106], [107], [108], [109], [110], [111], [112], [113], [114], [115], [116], [117], [118], [119], [120], [121], [122], [123], [124], [125], [126], [127], [128], [129], [130], [131], [132], [133], [134], [135], [136], [137], [138], [139], [140], [141], [142], [143], [144], [145], [146], [147], [148], [149], [150], [151], [152], [153], [154], [155], [156], [157]], of which 80 (58.8 %) reported a *C*_*llr*_ value in some way, either by giving an explicit (list of) value(s) or by plotting an ECE plot from which the *C*_*llr*_ value could be read. Out of the 80 publications that reported a *C*_*llr*_, 45 (56.3 %) also reported a *C*_*llr−min*_, making it possible to differentiate between discrimination and calibration error. As is shown in Fig. 1, the number of publications on (semi-)automated LR systems as well as the proportion of reported *C*_*llr*_ values differs widely per forensic expertise area. The various areas included were chosen based on a manual clustering of the selected literature taking into account common forensic expertise areas in literature and forensic practice. While the exact clustering is necessarily to some degree arbitrary, it is nevertheless helpful to illustrate patterns between various forensic sciences.

From Fig. 1 can be seen that by far the largest number of publications on (semi-)automated LR systems is in the area of speaker recognition. The proportion of publications reporting a *C*_*llr*_ in this area is also quite high (93.3 %). This makes sense given that speaker recognition is one of the most developed areas with respect to automated LR systems and is the forensic area in which the application of the *C*_*llr*_ was first proposed [9]. A relatively high proportion of publications report *C*_*llr*_ values in the area of materials and microtraces (81.0 %), which mainly includes publications on the individualisation of traces such as glass, paint and other substances. The area with the third largest number of publications is forensic DNA analysis. This is mainly due to the large number of probabilistic genotyping models that have been developed for the interpretation of DNA evidence (e.g. STRMix [111], EuroForMix [158], DNAxs [159], etc.). The null proportion of publications reporting a *C*_*llr*_ in this area (0 %) is striking. For stylometric analysis (100.0 %) and face comparison (85.7 %) the *C*_*llr*_ seems to be frequently used as well, but it should be noted that all publications found in stylometric analysis were written by the same author(s). In fingermark comparison, the *C*_*llr*_ seems to be less often used (50.0 %). Publications from the remaining expertise areas where less than 5 publications could be found were pooled together in a single “Other” category. For these categories, a bit less than half (44.0 %) of publications use the *C*_*llr*_ metric.

### 3.2 Proportion of publications reporting *C*_*llr*_ over time

**Fig. 2:**
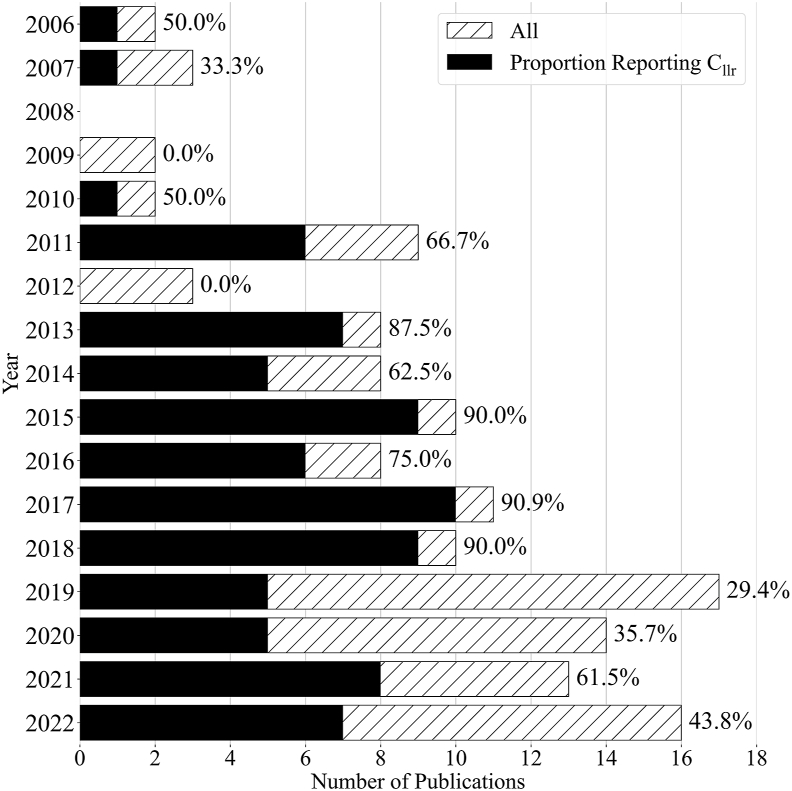
The number of publications on (semi-)automated likelihood ratio (LR) systems per year. The total number of publications per year is indicated by a dashed bar and the number of publications reporting *C*_*llr*_ values per year is indicated by a solid bar. The bars are overlapped, not stacked. The percentage of papers reporting a *C*_*llr*_ is printed on top of each bar.

**Fig. 3:**
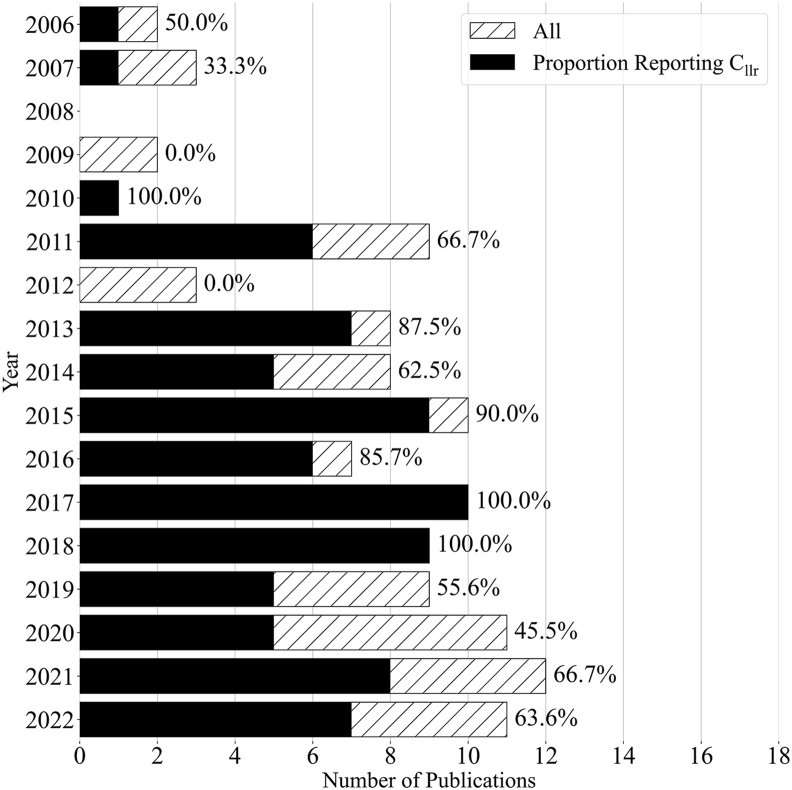
The number of publications on (semi-)automated likelihood ratio (LR) systems per year, excluding all publications in the area of DNA analysis. The total number of publications per year is indicated by a dashed bar and the number of publications reporting *C*_*llr*_ values per year is indicated by a solid bar. The bars are overlapped, not stacked. The percentage of papers reporting a *C*_*llr*_ is printed on top of each bar.

Besides looking at the differences between forensic areas, it is also interesting to look at the development of the application of the *C*_*llr*_ over time. Fig. 2 shows the number of publications on (semi-)automated LR systems and the proportion reporting a *C*_*llr*_ per year. There is a clear upward trend in the number of publications per year on (semi-)automated LR systems. Until 2018, no significant change is visible in the proportion of papers using the *C*_*llr*_ metric for evaluation. After 2018 however, the proportion of papers using the metric seems to be much smaller compared to the period before. This is due to some extent to an increased number of publications in the DNA analysis field in the last couple of years. When filtering out all DNA publications (Fig. 3), the difference between the periods becomes smaller, but remains present.

### 3.3 Comparison of *C*_*llr*_ values between forensic expertise areas

**Fig. 4:**
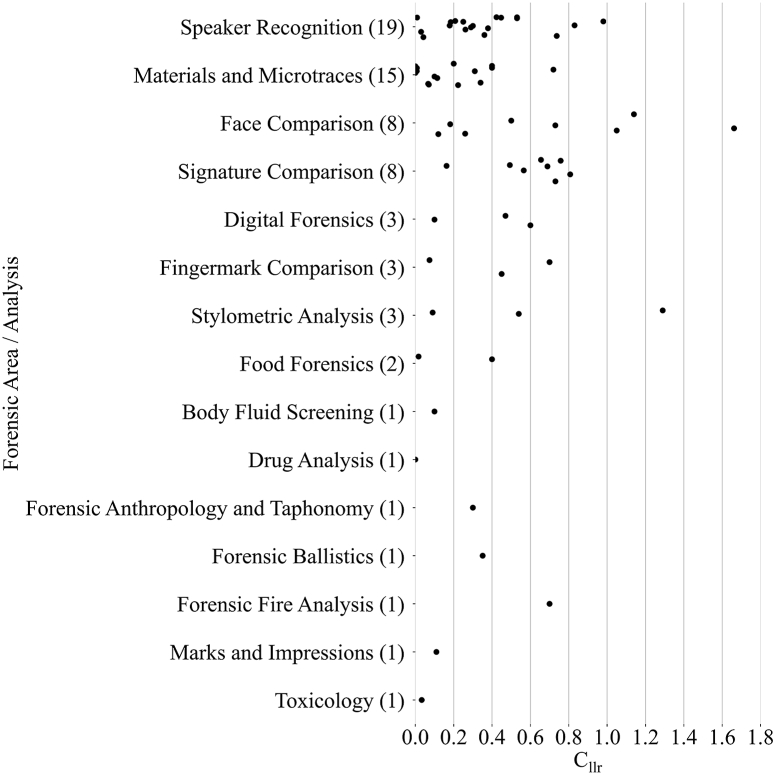
The best values per dataset (lower *C*_*llr*_ values indicate better performance) of selected forensically relevant *C*_*llr*_ values per forensic expertise area. The number in between brackets indicates the number of *C*_*llr*_ values plotted.

From the 80 publications on (semi-)automated LR systems that reported one or more *C*_*llr*_ values, we selected 95 forensically relevant *C*_*llr*_ values. Fig. 4 shows the distribution of forensically relevant *C*_*llr*_ values per forensic expertise area, only including the best values per unique dataset. Because different models evaluated on the same dataset can be compared and one would naturally pick the best model for application in practice, only values from the best models per dataset are shown. Note that Fig. 4 shows the best values per distinct dataset in a certain area, whereas Fig. 1 shows the number of unique publications per area. This explains why stylometric comparison, but not signature comparison, is shown separately in Fig. 1, even though the former shows fewer data points in Fig. 4. For the former, we found 8 publications with 3 *C*_*llr*_ values on distinct datasets, for the latter this was 3 publications with 8 *C*_*llr*_ values on distinct datasets.

From Fig. 4 it can be seen that there is quite a lot of variance in *C*_*llr*_ values within every area. Even in relatively well-defined areas such as speaker recognition and face comparison, *C*_*llr*_ values are distributed between [0.0002, 0.98] and [0.12, 1.662]. The values between the areas do follow, to some extent, the expected pattern. For example, we would expect much more discriminating results in materials and microtraces than in speaker recognition, and thus lower *C*_*llr*_ values. In contrast, for signature comparisons, we would expect higher *C*_*llr*_ values. This is clear from the figure, with *C*_*llr*_ values predominantly below 0.4 for the former, ranging across [0, 1] for speaker recognition and mainly around 0.6 for signature comparison. Literature does allow for drawing tentative conclusions on which *C*_*llr*_ values one may expect in general for a state-of-the-art system per area. We should be very careful in any conclusions though, as the values reported may fluctuate more because of the dataset queried than the method employed. Unfortunately, for many areas such as drug analysis and forensic ballistics, only one or two *C*_*llr*_ values are available, making it hard to draw any conclusions. This also makes it more difficult to observe any clear pattern in *C*_*llr*_ values in general.

## 4 Discussion

There are slight differences visible between *C*_*llr*_ values reported in different forensic expertise areas, with fields that generally provide higher discriminating power generally showing lower *C*_*llr*_ values. However, the abundance of diverse datasets used for evaluation and the relative scarcity of *C*_*llr*_ values reported make it difficult to distinguish any clear patterns. This is a disappointing finding, as the current state of the literature thus not allows for further intuition on what constitutes a ‘good’ *C*_*llr*_. To address the noise caused by evaluation on these diverse datasets, different systems should be evaluated on standard, forensically relevant, benchmark datasets which would allow for a much more direct comparison between systems. The set of *C*_*llr*_ reference values resulting from such evaluation can serve as a valuable resource for assessing the quality of (semi-)automated systems being developed in the future.

There is no objective system for assessing whether a dataset is ‘forensically relevant’. For this study, we subjectively assessed whether evaluations were conducted under circumstances resembling casework conditions. In this way, we aimed to get at least a global view of *C*_*llr*_ values that can be expected for a system in practice, while mitigating the confounding impact of unrealistic setups. Nevertheless, what constitutes forensic relevance remains a subjective decision that may differ per jurisdiction or organization.

Even though the number of publications on (semi-)automated LR systems has been increasing since 2006, the use of the *C*_*llr*_ metric has remained relatively constant. It is challenging to explain this phenomenon, but it is plausible that the stability of the metric usage can be more attributed to an existing user base that has been publishing more over the years, rather than widespread adoption of the metric across the field.

In addition, it is worth noting the seemingly non-existent use of *C*_*llr*_ in forensic DNA analysis. Especially, since its use seems particularly well suited for forensic DNA comparison, given the well-established Bayesian reasoning methodology in the field. One possible reason is the fact that there are cases where the *C*_*llr*_ may not be so relevant. For instance, in cases without contamination, mixtures, dropout, etc., and with abundant samples, DNA LR values present high discriminating power in general. As the *C*_*llr*_ is an empirical measure, generating a sufficiently large number of LR values is necessary to obtain statistically stable *C*_*llr*_ values, and in cases with very high discriminating power, all *C*_*llr*_ values may be numerically zero or very close to zero. This reduces the usefulness of the metric. Another reason could be that, because the metric has historically been rarely used in the field, subsequent publications may also overlook it.

Although much attention in forensic DNA analysis is devoted to validation, there is little emphasis on specific metrics [[160], [161], [162], [163]]. Usually well-known metrics are employed, e.g. ROC, AUC, false positive rates (given a certain LR threshold), or rates of misleading evidence [[164], [165], [166]]. We have not seen metrics specifically for calibration, even though the concept is certainly recognized in the field [138,167,168]. There thus may be a role to play for strictly proper scoring rules, which can comprehensively indicate the performance of an LR system.

## 5 Conclusions

The aim of this review was to gain insight into what *C*_*llr*_ values can be expected for state-of-the-art systems in forensic sciences, and to investigate the current application of the log-likelihood ratio cost (*C*_*llr*_) in the evaluation of (semi-)automated likelihood ratio (LR) systems. While no claim is made that all existing relevant literature was found, considerable effort was put into getting an as complete picture as possible. We found that the use of the *C*_*llr*_ metric heavily depends on the field, with the metric being conspicuously absent in the forensic DNA analysis field. In addition, we found that the number of publications on (semi-)automated LR systems has increased over the years, but the use of the *C*_*llr*_ metric has remained more constant over time. The results do not show a clear pattern for what *C*_*llr*_ values can be expected and values vary a lot between forensic expertise areas, types of analyses, and datasets. Hence, we cannot establish a clear range of variation in general to assess the goodness of a system computing LR values. We set out to investigate if we could get a feeling of what a ‘good’ *C*_*llr*_ value is. We are not quite there yet. A path forward may be provided by benchmarks, i.e. datasets and evaluation protocols publicly available. Such benchmarks are standard practice in many fields of machine learning, and also used in several forensic fields [169], and allow for a much more direct comparison between systems. Given the trend that the number of LR systems developed and used increases yearly, having the ability to properly evaluate them will be increasingly important. Thus, setting up and curating such benchmark datasets, and establishing suitable evaluation protocols, is an investment in the future of the field.

## Data availability

The data that support the findings of this study as well as the code used to analyze the data are available at https://github.com/NetherlandsForensicInstitute/cllr-review.

## Funding

This research did not receive any specific grant from funding agencies in the public, commercial, or not-for-profit sectors.

## CRediT authorship contribution statement

**Stijn van Lierop:** Writing – original draft, Investigation, Data curation. **Daniel Ramos:** Writing – review & editing. **Marjan Sjerps:** Writing – review & editing, Supervision. **Rolf Ypma:** Writing – review & editing, Supervision, Conceptualization.

## Declaration of competing interest

The authors declare that they have no known competing financial interests or personal relationships that could have appeared to influence the work reported in this paper.

## Figures and Tables

**Table A.3:** Summary of all literature per forensic expertise area and type of analysis. If a *C*_*llr*_ is reported, information about the evaluation dataset is also given.

Ref.	Cllr (Cllr-min)	Dataset
Forensic Biometrics – Speaker Recognition		
[9]	0.379 (0.366)	Detection scores provided by three speaker verification teams.
[10]	0.25 (0.21)	NIST 2006 SRE (608 speakers) and NIST SRE 2005
[34]	0.068	Laboratory-quality audio dataset of 27 Australian male speakers by Yuko Kinoshita.
[36]	0.1845 (0.16)	NIST SRE Data, 2008 data for training and 2010 data for evaluation.
[35]	0.98 (0.43)	Shenyang China Male Speaker set, 64 male speakers of standard Chinese aged between 19 and 23.
[46]	0.042	60 female speakers from Northeastern China
[47]	0.3	GFS1 Corpus (German police male telephone tap recordings) containing 39 offender recordings, 21 suspect recordings and 49 reference population recordings.
[44]	0.009	297 male speakers selected to give geographical representative coverage of Japan.
[49]	0.375	NIST SRE Data, 2008 data for training and 2010 data for evaluation
[48]	0.496	Archive of chatlog messages of pedophiles (115 authors)
[14]	0.53 (0.47)	NIST HASR Dataset (150 comparisons)
[53]	0.447 (0.405)	4188 intercepted telephone speech by police in Dutch, Moroccan, Arabic, Berber and Turkish files with speech from 604 different speakers.
[62]	0.1092	background population: 50 speakers with similar background, reference population: 20 speakers with same background as speakers in background population.
[63]	0.29	26 young Australian female speakers, calculated 22 same speaker LRs and 231 different speaker LRs.
[66]	0.53	Telephone recordings of pairs of male speakers from a database of Australian English voice recordings designed for forensic casework.
[70]	0.179 (0.154)	MARP corpus containing 60 speakers (35 male, 25 female).
[71]	0.365	forensic_eval_01 data with 100 speakers in reference population.
[73]	0.423	Australian English Male of 231 Speakers designed for Forensic Research.
[79]	0.03	HKMTR database containing 23 speakers
[80]	0.36	136 Australian Female Speakers
[82]	0.449 (0.148)	forensic_eval_01 data, used 423 recordings from 105 speakers.
[84]	0.5	Corpus of spontaneous Japanese, 118 speakers (40 test, 39 background, 39 development), segments of around 10 min containing audio from presentations and mock conversations.
[86]	0.674 (0.658)	forensic_eval_01 data 105 speakers (train) and 61 speakers (test).
[90]	0.261	Questioned speaker telephone calls, known speaker police interviews, 111 SS and 9720 DS scores, 105 recordings training and 61 testing.
[93]	0.5192	forensic_eval_01 data, 423 recordings from 105 speakers for training, 223 recordings from 61 speakers for testing.
[94]	0.593 (0.198)	forensic_eval_01 data, 100 speakers in reference population.
[95]	0.83	DyVis Corpus, southern British male speakers, 97 SS and 4656 DS comparisons, 60 s mobile telephone calls.
[98]	0.208 (0.098)	forensic_eval_01 data
[100]	0.246 (0.189)	forensic_eval_01 data (223 recordings from 61 speakers).
[99]	0.255 (0.124)	forensic_eval_01 data
[102]	0.737	UCLA Speaker Variability Database, 103 female and 105 male speakers, 3 recordings per speaker.
[110]	Not reported	
[156]	Not reported	
Forensic Biometrics – Fingermark Comparison		
[170]	Not reported	
[28]	Not reported	
[45]	Not reported	
[50]	Not reported	
[52]	0.074 (0.063)	58 fingermarks real case data for evaluation, 25000 simulated fingermarks of 200 individuals for training.
[76]	0.165 (0.11)	Development set for training and validation casework fingerprint dataset for testing.
[77]	0.7	Biometric Collection of People 2008
[81]	0.45 (0.35)	NFI Casework dataset + Simulated dataset + Printcards from Dutch national database (HAVANK2).
Forensic Biometrics – Face Comparison		
[90]	0.104	XM2VTS database with 225 faces (8 images per face).
[97]	0.0935 (0.036)	XM2VTS and Face-3D Datasets
[115]	0.00177	National mugshot database, 51562 records, 9000 facial comparisons.
[117]	0.43	2011 ENFSI Face Proficiency Tests
	0.5	2017 ENFSI Face Proficiency Tests
	0.57	2013 ENFSI Face Proficiency Tests
	1.23	2012 ENFSI Face Proficiency Tests.
[141]	Not reported	
[146]	0.26	FaceScrub database
[144]	0.12	2011 ENFSI Face Proficiency Tests
	1.05	2012 ENFSI Face Proficiency Tests
	0.182	2013 ENFSI Face Proficiency Tests
	0.687	2017 ENFSI Face Proficiency Tests
	0.73	2018 ENFSI Face Proficiency Tests
	1.14	2019 ENFSI Face Proficiency Tests
	1.662	2020 ENFSI Face Proficiency Tests.
Forensic Biometrics – Stylometric Analysis		
[78]	1.29 (0.96)	NUS SMS Corpus (Singapore, India and US messages mostly).
[55]	0.54	Archive of chatlog messages of pedophiles (115 authors).
[83]	0.09 (0.05)	Archive of chatlog messages of pedophiles (115 authors).
[85]	0.683	Archive of chatlog messages of pedophiles (115 authors).
[116]	0.7	Amazon Product Data Authorship Verification Corpus.
[130]	0.66703	Amazon Product Data Authorship Verification Corpus.
[148]	0.5395 (0.51987)	Amazon Product Data Authorship Verification Corpus, 2157 authors, 3 document lengths (700, 1400 and 2100 words).
Forensic Biometrics – Handwriting Comparison		
[41]	Not reported	
[43]	Not reported	
[171]	Not reported	Created handwriting samples similar to casework, 7196 responses from 86 participants on 180 distinct QKsets.
[140]	Not reported	
[143]	Not reported	
Forensic Biometrics – Signature Comparison		
[37]	0.493 (0.238)	Dataset of Dutch online real or simulated (forged) signatures
	0.565 (0.351)	Dataset of Chinese online real or simulated (forged) signatures
	0.730 (0.573)	Dataset of Dutch offline real or simulated (forged) signatures
	0.758 (0.693)	Dataset of Chinese offline real or simulated (forged) signatures
[65]	0.655 (0.021)	Real life offline signatures from Italian university employees and students over a span of several years + forgeries, 628 total (250 training + 478 testing)
	0.689 (0.052)	240 real Bengali offline signatures from 10 contributors + forgeries from different parts of West-Bengal (India) (120 training and 420 testing)
	0.807 (0.466)	750 German online signatures (300 training + 450 testing) gathered by researchers with digital pen.
[89]	0.1629 (0.1433)	Chinese Signature Database with 1654 signatures.
Forensic Biometrics – Earmark Comparison		
[42]	Not reported	
Forensic Biometrics - Gait Analysis		
[39]	Not reported	
Forensic Biology – DNA Analysis		
[32]	Not reported	
[75]	Not reported	
[87]	Not reported	
[96]	Not reported	
[104]	Not reported	
[106]	Not reported	
[109]	Not reported	
[107]	Not reported	
[172]	Not reported	
[112]	Not reported	
[113]	Not reported	
[114]	Not reported	60 mixed DNA profiles of up to 5 contributors per mixture.
[153]	Not reported	
[125]	Not reported	
[127]	Not reported	
[128]	Not reported	
[138]	Not reported	
[173]	Not reported	
[150]	Not reported	
[151]	Not reported	
[152]	Not reported	
Forensic Biology - Body Fluid Identification		
[131]	0.1	Laboratory mixture data.
Materials and Microtraces – Glass Classification		
[33]	0.07 (0.01)	SEM-EDX Glass Database of 278 objects (79 containers and 199 float glass samples).
Materials and Microtraces – Glass Individualisation		
[31]	0.31 (0.26)	Database of UK and Polish window- and container glass SEM-EDX measurements.
[13]	0.72 (0.42)	Poland SEM-EDX glass dataset containing 165 glass objects (87 car windows and 78 building windows).
[60]	0.043 (0.026)	Raman micro spectroscopy database of solid car paint samples coming from 25 solid blue car paints, each sample measured at least three times.
[61]	0.2 (0.15)	SEM-EDX measurements from Institute of Forensic Technology Krakow.
[88]	0.0081 (0.0045)	Glass database of 979 glass particles from different sources of crime scene glass (known source) from about 10 years of casework at the NFI.
[90]	0.007	LA-ICP-MS glass sample data from over 10 years of casework, 659 sources for training and 320 sources for testing.
[174]	0.067 (0.017)	BKA glass database as background with FIU glass database for testing.
[101]	Not reported	
[139]	0.29 (0.079)	BKA glass database as background with FIU glass database for testing.
[145]	0.12 (0.1)	Swedish SEM-EDX Glass dataset containing 380 glass samples from Swedish casework measured in 5 replicates.
Materials and Microtraces – Ink Individualisation		
[29]	Not reported	
[68]	0.1833 (0.0899)	10 samples each of 40 different ink sources
Materials and Microtraces – Paint Individualisation		
[30]	Not reported	
Materials and Microtraces – Car Fuel Individualisation		
[51]	0.1 (0.01)	Repeated samples from 15 different petrol stations in The Hague.
Materials and Microtraces – Gasoline Individualisation		
[129]	0.028 (0.024)	GC-MS diesel oil dataset, 158 diesel oil samples from Swedish gas stations and oil refineries between 2015 and 2020
Materials and Microtraces – Car Paint Individualisation		
[59]	0.19 (0.1)	Raman micro spectroscopy database of metallic car paint samples from 30 metallic blue car paints, each sample measured at least three times.
[59]	0.07 (0.03)	Raman micro spectroscopy database of solid car paint samples from 25 solid blue car paints, each sample measured at least three times.
[60]	0.227 (0.147)	Raman micro spectroscopy database of metallic car paint samples from 30 metallic blue car paints, each sample measured at least three times.
[69]	0.0002 (0.0002) 0.0005 (0.0005) 0.223 (0.078)	Raman micro spectroscopy database of metallic car paint samples from 30 metallic blue car paints, each sample measured at least three times Raman micro spectroscopy database of solid car paint samples from 25 solid blue car paints, each sample measured at least three times Raman spectroscopy database of polypropelene spectra.
[68]	0.1756 (0.1533)	7 measurements each from 36 different acrylic car paint sources.
Forensic Ballistics – Cartridge Case Comparison		
[56]	Not reported	
Forensic Ballistics – Pattern Matching		
[121]	Not reported	2D measurements from 400 cartridge cases of 200 9 mm Glock pistols 3D measurements from 400 cartridge cases of 200 9 mm Glock pistols.
[123]	Not reported	
[124]	Not reported	
[122]	Not reported	
[108]	Not reported	
[175]	Not reported	
[176]	Not reported	
[177]	Not reported	
[147]	0.351	3D digital images of the bases of approximately 3000 fired cartidge cases.
Marks and Impressions – Shoemark Comparison		
[38]	Not reported	
Marks and Impressions – Tape Comparison		
[57]	Not reported	
[103]	0.11 (0.08)	LA-ICP-MS data for 90 electrical tapes
Marks and Impressions – Tape Comparison		
[178]	Not reported	
Forensic Drug Analysis – Drug Individualisation		
[40]	Not reported	
[58]	0.00006	GC-MS chemical impurity profiles of MDMA tablets collected by the Netherlands Forensic Institute (NFI) between 2004 and 2011 from 1372 batches, 20 pills each.
Forensic Drug Analysis – Activity Level Drug Analysis		
[142]	Not reported	
Toxicology – Alcohol Abuse Prediction		
[72]	0.033 (0.033)	Database of regional antidoping and toxicology center from 125 subjects (118 males and 7 females).
[120]	0.08	Database of regional antidoping and toxicology center containing 125 subjects (118 males and 7 females).
Food Forensics – Fraud		
[54]	0.4	178 wine samples from 3 brands of Italian wines
[64]	0.017 (0.017)	Olive oil dataset of 572 Italian olive oils described by fatty acid content.
Materials and Microtraces – Car Plastics Individualisation		
[91]	0.4 (0.3)	22 objects from 22 different vehicles/plastic containers 63 SS scores and 1890 DS scores.
Materials and Microtraces – Gunshot Residue Analysis		
[155]	Not reported	
[149]	0.34	1953 stubs from 210 criminal cases, 1557 same-shot pairs and 859.515 different-shot pairs.
Materials and Microtraces – Geolocation		
[92]	0.4 (0.25)	104 wolframite samples (5327 grains) from 45 different mines from 10 countries worldwide.
Explosions and Explosives – Explosive Individualisation		
[27]	Not reported	
[67]	Not reported	
[74]	Not reported	
Forensic Anthropology and Taphonomy – PMI Estimation		
[105]	Not reported	
Forensic Anthropology and Taphonomy – Stature Estimation		
[119]	Not reported	
Forensic Anthropology and Taphonomy – Sex Estimation		
[132]	0.3	Bartholdy et al. Dataset [179]
[133]	0.42 Not reported	FaceScrub database, 100.000+ images of more than 250 different males and 250 different females SCFace Databse.
[118]	Not reported	
Digital Forensics – Camera Identification		
[126]	Not reported	
Digital Forensics – Route Determination		
[157]	0.1 (0.08)	Set of distances measured using iPhone health apps using different iPhones and wearing pace and locations (1015 training set, 677 test set).
Digital Forensics – Mobile Phone Pairing		
[135]	0.47	Call records from 22 phone users (15 had two phones).
Digital Forensics – Camera Identification		
[134]	0.6 (0.57)	Vision Dataset, 34 different devices, around 7000 images, 223 non-stabilized videos and 190 stabilized videos.
[154]	Not reported	
Forensic Fire Analysis – Fire Debris Classification		
[136]	0.7 (0.58)	Fire debris GC-MS dataset, 492 ground truth IL and 299 ground truth SUB samples.
Forensic Odour Evidence – Hand Odour Identification		
[137]	Not reported	
